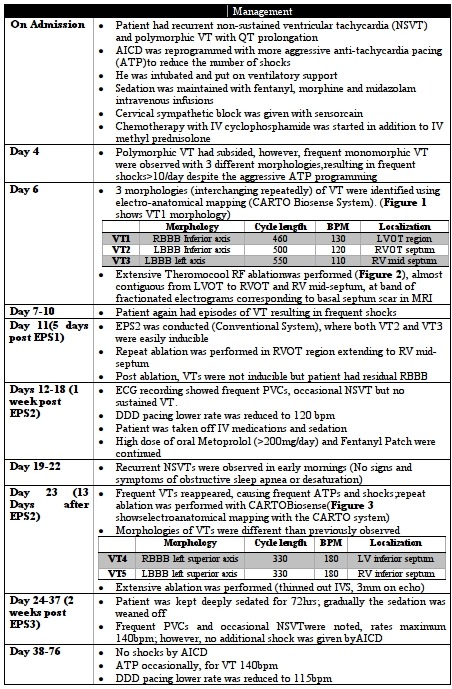# Ventricular Tachycardia Storm in Cardiac Sarcoidosis: A 76-Day-ICU-Nightmare

**DOI:** 10.1016/s0972-6292(16)30567-8

**Published:** 2012-12-02

**Authors:** Ajay M Naik, Sizan B Patel

**Affiliations:** Department of Cardiology, CIMS Hospital, Ahmedabad, Gujarat, India

**Keywords:** Cardiac Sarcoidosis, Ventricular Tachycardia Storm, Implantable Cardioverter Defibrillator, Radio-Frequency Ablation

## Abstract

Ventricular Tachycardia (VT) is a life threatening complication in a patient with Cardiac Sarcoidosis. The management becomes extremely challenging when it is refractory to traditional anti-arrhythmic drugs. Herein, we describe a case where a 33-year-old patient with VT storm, with an implantable cardioverter defibrillator (ICD), was managed by medications, sedation, ventilator support and multiple Radio-Frequency (RF) ablation procedures over 76-days ICU stay period.

## Introduction

Sarcoidosis is a multisystem disease of unknown etiology. With the advent and frequent use of current diagnostic modalities, cardiac involvement in sarcoidosis is now frequently diagnosed. Cardiac sarcoidosis related arrhythmia can be very difficult to manage. VT is in fact one of the most frequent arrhythmias encountered in cardiac sarcoidosis and can be present in up to 23% of the patients [1]. Because of increased awareness of the risk of sudden cardiac death and early ICD implantation, cardiac sarcoidosis patients can survive to present with VT storm, which is not uncommonly resistant to standard anti-arrhythmic drugs. We report a case with a patient diagnosed with cardiac sarcoidosis, suffering from refractory ventricular tachycardia (VT) resulting in frequent shock therapies from ICD, successfully stabilized by multiple RF ablation procedures over two and half months of ICU care.

## Case Presentation

A 33-year-old man was incidentally detected to have Premature Ventricular Contractions (PVCs) during annual health check-up. There were no other remarkable features in electrocardiogram (ECG) or echocardiogram (LVEF 60% and no Right Ventricular disease) in January 2011. Following month, the patient got admitted with palpitations and presyncope, and diagnosed with sustained VT, which was alleviated with direct current (DC) cardioversion, IV Amiodarone and Beta blockers. Detailed investigations were performed including contrast enhanced computed tomography (CECT) of chest, cardiac MRI, followed by endoscopic ultasonography guided fine needle aspiration cytology (FNAC) which were all suggestive of sarcoidosis, therefore steroid immunosuppressive therapy was started at this time.

**Figure 1:**
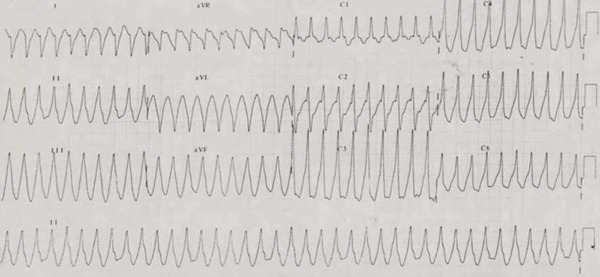
12 Lead Electrocardiogram showing VT morphology 1

**Figure 2:**
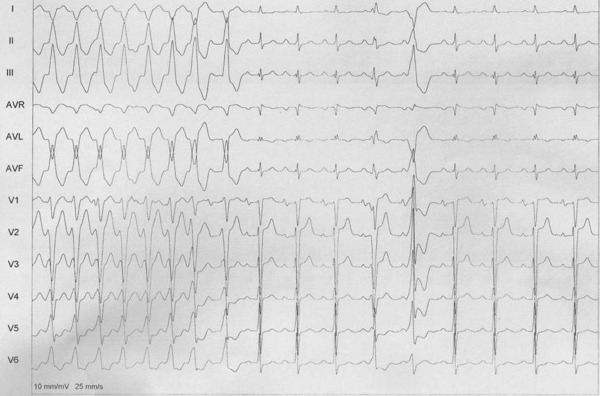
VT2 termination during RF ablation

**Figure 3:**
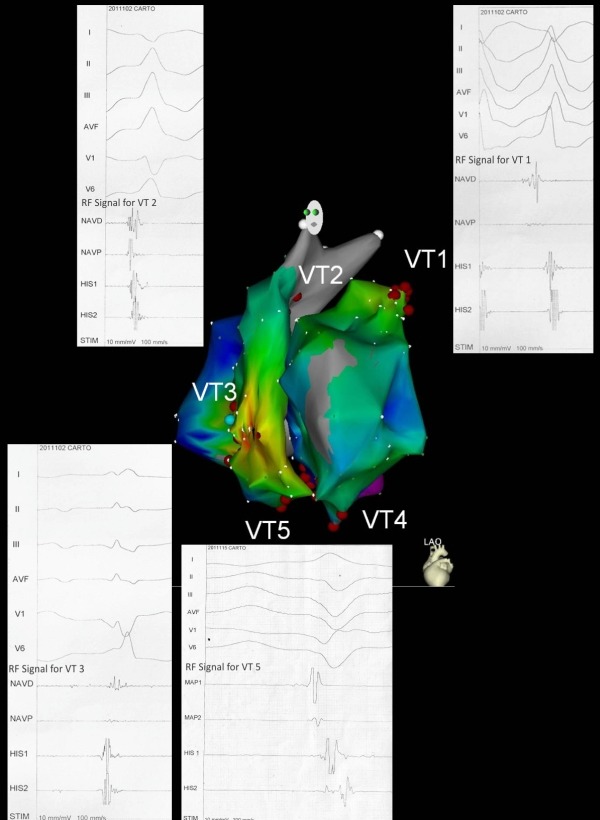
Electroanatomical mapping with the CARTO system, composite RV and LV pictures showing sites of ablation of 5 VT morphologies

In June 2011, the patient once again presented with sustained rapid monomorphic VT and DC cardioversion was performed. Echocardiogram demonstrated left ventricular ejection fraction (LVEF) of 15%, LVDd of 64mm, LVDs of 58mm, and grade II mitral valve regurgitation. Dual chamber implantable cardioverter defibrillator (ICD) was implanted and patient was treated with beta blockers, Lidocaine and Amiodarone. Despite anti-arrhythmic drugs (AAD), the patient had recurrent VT, which was successfully treated by ICD with 12 shocks in the duration of 24 hours. He was intubated, placed on ventilatory support and was kept under sedation. In addition to AAD, IV Magnesium sulphate and Metoprolol infusions were started. The patient was weaned off the ventilator after 3 days and transferred to our institute via airplane; he received shocks from ICD even during the transfer. Management during ICU stay is outlined below (Table 1; Figure 1,2,3).

**Figure 4:**
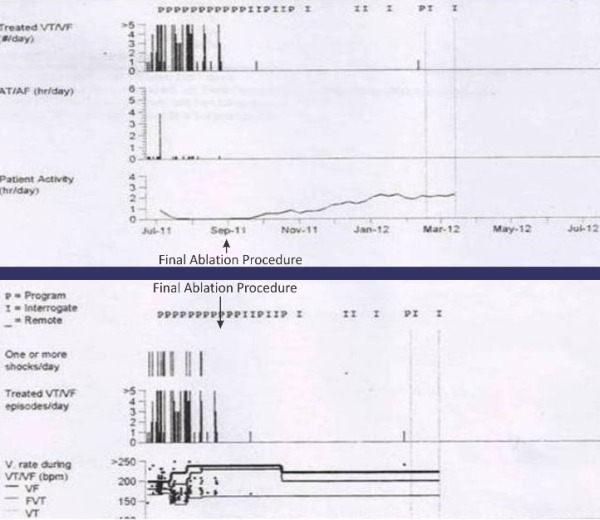
Cardiac compass reports showing marked reduction in VT/VF episodes and increase in patient activity following final ablation procedure

Patient was observed and finally discharged after 76 day of ICU stay. The echocardiogram findings demonstrated LVEF of 15%, dilated Left Ventricle and thinned out myocardium. During the follow-up (6 months post discharge), patient was performing reasonably well, with LVEF of 25%, less frequent non-sustained VT and only one shock from ICD. (Figure 4 shows cardiac compass trends of the patient).

## Discussion

Sarcoidosis is a multisystem granulomatous disease of unknown etiology characterized by the presence of noncaseating granulomas in the involved organs. Cardiac involvement in patients with sarcoidosis is getting vastly recognized and is associated with poor prognosis. The clinical sequelae of cardiac sarcoidosis range from asymptomatic conduction abnormalities to fatal ventricular arrhythmias, depending upon the location and extent of granulomatous inflammation. Sarcoid granulomas in the ventricular myocardium serve as foci for abnormal automaticity and cause changes in the ventricular activation and recovery process, which explains the reentry mechanism that is thought to lead to VT, the most frequent arrhythmia noted in cardiac sarcoidosis [1]. Some patients have VT and concomitant intermittent atrioventricular block, a combination that more typically suggests cardiac Sarcoidosis [2].

Sarcoidosis that involves the heart warrants prompt therapy with steroids, immunosuppressive agents, or both [3]. Steroids are believed to be capable of attenuating the inflammatory response and slowing down subsequent fibrosis, but it is less effective in preventing further arrhythmia as it is shown to be different inducibility of VT between the active and inactive phases of sarcoid heart disease in an EP study [1]. Management of arrhythmias in cardiac sarcoidosis is difficult and effective control of VT often is not achievable by a single method of therapy. Current studies recommend cautious use of AAD therapy for ventricular tachycardia in patients with cardiac sarcoidosis as it may result in high rate of recurrence or sudden death. Amiodarone use in this group of patients may be limited by the occurrence of pulmonary complications which are often difficult to distinguish from pulmonary sarcoidosis. Beta Blockers also have shown to increase the incidence of heart blocks in patients with cardiac sarcoidosis and it becomes more concerning especially if patients have underlying conduction blocks on surface ECG prior to the initiation of therapy [4]. Immunosuppressive therapies are recommended as a steroid sparing strategy in the management of sarcoidosis and can be beneficial in controlling arrhythmia. Treatment with an ICD along with anti-arrhythmic therapy is mandatory in sarcoidosis patients with refractory VT, who are at risk of sudden death. Prophylactic ICD placement is also recommended in patients with diagnosed sarcoidosis who develop VT during exercise electrocardiography or Holter monitoring [5]. Implantable defibrillators can terminate ventricular arrhythmias and prevent sudden death but do not prevent these arrhythmias from recurring. Ablation should be considered as an option in those patients with recurrent VT that are unresponsive to anti-arrhythmic and immunosuppressive agents. RF ablation is very effective in decreasing or eliminating completely episodes of VT in a patient with cardiac sarcoidosis. Data from multicenter registry showed that VT burden was reduced more than 98% in the first 3 months post ablation in patients with cardiac sarcoidosis; the mechanism of VT in virtually all patients was re-entry [6]. Though, it is usual to perform multiple RF ablation in such patients, VT circuits can be changing as inflammation waxes and wanes.

In conclusion, in the present case of a patient with sarcoidosis and cardiac involvement, multiple sessions of radiofrequency catheter ablation of VT refractory to immunosuppressive therapy and traditional anti-arrhythmic drugs, were effective in controlling the VT storms. Survival after a life-threatening illness and prolonged hospitalization in a distant city (representing major financial, emotional and social stress on the family) was especially gratifying as the patient was able to resume a productive career.

## Figures and Tables

**Table 1:**